# A Hybrid Method for Endocardial Contour Extraction of Right Ventricle in 4-Slices from 3D Echocardiography Dataset

**DOI:** 10.1155/2014/207149

**Published:** 2014-10-12

**Authors:** Faten A. Dawood, Rahmita W. Rahmat, Suhaini B. Kadiman, Lili N. Abdullah, Mohd D. Zamrin

**Affiliations:** ^1^Department of Computer Science, College of Science, University of Baghdad, Baghdad, Iraq; ^2^Department of Multimedia, Faculty of Computer Science and Information Technology, UPM, 43400 Serdang, Selangor, Malaysia; ^3^Department of Anaesthesiology, Institute Jantung Negara (IJN) Hospital, Jalan Tun Razak, 50400 Kuala Lumpur, Malaysia; ^4^Department of Surgery, Heart and Lung Centre, HUKM, National University of Malaysia, 56000 Cheras, Kuala Lumpur, Malaysia

## Abstract

This paper presents a hybrid method to extract endocardial contour of the right ventricular (RV) in 4-slices from 3D echocardiography dataset. The overall framework comprises four processing phases. In Phase I, the region of interest (ROI) is identified by estimating the cavity boundary. Speckle noise reduction and contrast enhancement were implemented in Phase II as preprocessing tasks. In Phase III, the RV cavity region was segmented by generating intensity threshold which was used for once for all frames. Finally, Phase IV is proposed to extract the RV endocardial contour in a complete cardiac cycle using a combination of shape-based contour detection and improved radial search algorithm. The proposed method was applied to 16 datasets of 3D echocardiography encompassing the RV in long-axis view. The accuracy of experimental results obtained by the proposed method was evaluated qualitatively and quantitatively. It has been done by comparing the segmentation results of RV cavity based on endocardial contour extraction with the ground truth. The comparative analysis results show that the proposed method performs efficiently in all datasets with overall performance of 95% and the root mean square distances (RMSD) measure in terms of mean ± SD was found to be 2.21 ± 0.35 mm for RV endocardial contours.

## 1. Introduction 

The importance of the RV systolic function has been recognized in many cardiovascular diseases and cardiac surgery. In clinical practice, echocardiography is widely used, noninvasive, and cost-effective technique. It could be the best modality choice to give a better understanding of the right ventricular morphology and function analysis [1]. The RV anatomical complexity and its geometrical asymmetric shape make automatic extraction of RV endocardial contour a difficult task, and, thus, it is still a great challenge for most researchers, especially in the field of computer aided diagnosis (CAD) [2–5]. An accurate quantification analysis of the RV function is an important diagnostic parameter and largely depends on how the RV cavity successfully segmented based on endocardial contour extraction in complete cardiac cycle. For many years, most of the studies focused on the assessment analysis of the left ventricular (LV) morphology and function, which overshadowed that of RV analysis [6]. Currently, in most medical imaging applications, segmentation is one of the essential steps used for clinical measurement, feature extraction, and 3D visualization [7, 8]. Moreover, it is one of the important challenging issues for most researchers in the field of diagnostic of the heart abnormality. In most reviewed literatures, a number of RV automatic and semiautomatic segmentation methods have been presented for MRI [9–13]. The important objective in most methods is to develop the segmentation process by distinguishing the image into distinct regions (i.e., ventricular cavity and myocardial wall tissue) [14, 15]. In echocardiography images, thin walls, heavy trabeculation, and no simple geometric shape that approximates to the RV are factors that make determining the cardiac structure of the RV cavity very difficult. Due to these characteristics, not many researches have attempted to tackle this topic [16, 17], while a wide variety of LV segmentation techniques have been proposed as automatic and semiautomatic [18–21]. Nandagopalan et al. [22] proposed an automatic approach for segmentation and ventricular border detection that combined *k*-means clustering and active contour model. In the proposed approach, the ventricles cavities segmentation of LV and RV are based on many calculations. A new automated segmentation technique has been presented by Katouzian et al. [11] for detecting endocardial and epicardial borders for LV and RV in magnetic resonance imaging (MRI).

The anatomical complexity and asymmetric shape of RVs make accurate automatic detection of endocardial contours through complete cardiac cycle an extremely challenging task. Over the years, a considerable amount of quantitative echocardiographic information has been made public such as cardiac anatomy, chamber diameter and volume, wall thickness, valve, and ejection fraction. The interest to the practicing physician is that if the endocardial border has been accurately approximated then the chamber cross-sectional area can be estimated. Therefore, in most literatures, many clinical parameters have been developed to determine the RV function using 2D echocardiography, such as TAPSE, Tei-Index, strain, and speckle tracking [23, 24]. Most approaches require delineation of the inner surface (endocardial contour) of the RV cavity area, which is manually selected by expert for accurate measurement. However, current procedures are time consuming, often requiring several minutes to obtain an accurate estimation of an endocardial contour. Clinically, in many cases, time is of the essence in assessing the status of patients, where decisions on what medical procedure to take could be made without sufficient information [25]. Therefore, automation is desirable so that the appropriate information can be obtained faster (in this case the RV boundaries). Accordingly, in many studies, the endocardial surface boundary detection is found semiautomatically for LVs, and the same approach is still used for RVs to aid the volume calculation [26].

Recently, the developments in real-time 3D echocardiography (RT3DE) have allowed RV images to be viewed more comprehensively, where views of all portions of its asymmetric shape is made possible, allowing accurate assessment in a reproducible and rapid manner [27]. In the past, acquisition of RV echocardiographic data was very difficult due to its anterior position, complex geometry, and thin wall with prominent trabeculations. In the literature, RT3DE is reported to determine RV volumes and ejection fraction (EF) [28–32]. To the best of our knowledge, automated contour extraction of right ventricular endocardial has not been attempted for 3D echocardiography. The main goal of the proposed method is to segment the RV cavity area based on endocardial contour extraction over a complete cardiac cycle using 4-slices of 3D echocardiography. Assuming that the RV endocardial contour has been extracted accurately, this automated segmentation process can be a very useful diagnostic tool for assessing the RV systolic function, both qualitatively and quantitatively. In addition, the automated extraction of the RV endocardial contour would improve the reliability of the quantitative analysis by eliminating the subjectivity of manual tracing. In particular, it could be a significant prognostic factor for regional ventricle abnormality, congenital heart disease, and even cardiac surgery.

## 2. Echocardiography Dataset Preparation

**Figure 1:**
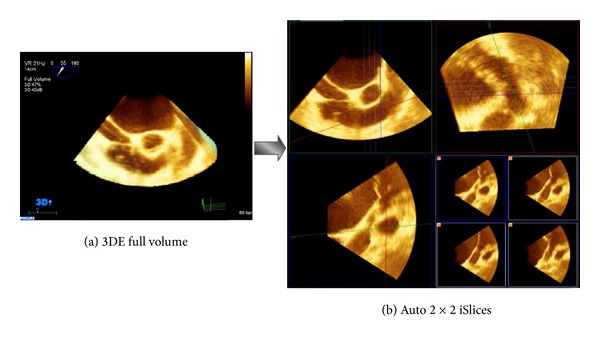
3D echocardiography dataset preparation including 3DE full volume acquisition (a) and 4-slices encompassing the RV of long-axis view using MPR mode in 3D QLAB software (b).

Echocardiography is the application of diagnostic ultrasound imaging to the heart. It has been received in the evaluation of cardiac disease and in characterizing the structure and function of the heart. One advantage over other imaging modalities is its ability to generate real-time images of anatomy without using ionizing radiation. Conventionally, RV echocardiographic data acquisition is very challenging due to its anterior position, complex geometry, and morphology with prominent trabeculations. In our work, all datasets of 3D echocardiography were obtained from a Malaysian hospital, IJN (National Heart Institute). Four stages of dataset preparation process are required; firstly, a live 3D full volume dataset encompassing the RV were acquired using a matrix array X2-7t transducer (TEE). In the second stage, all the acquired 3D datasets were transferred from the online medical system “Philips” directly to an “Xcelera” server in the offline workstation by running QLAB software. In the third stage, the 3D RV full volume dataset was viewed as orthogonal slices using the “MPR” mode (multiplanar reconstruction) and 3D quantification (3DQ) plug-in. Hence, 4-slices from 3D full volume encompassing RV in long-axis view are identified based on inflow-outflow view using “2 × 2 iSlices” plug-in. Finally, these 4-slices were stored individually as videos with complete cardiac cycle in AVI format. Then, each video was converted to a fixed number of frames (*F*1, *F*2, *F*3, …, *Fn*) according to frame rate, where each frame is represented as a BMP image. The 4-slices dataset preparation from 3D echocardiography full volume is presented in Figure 1.

## 3. The Proposed Method

**Figure 2:**
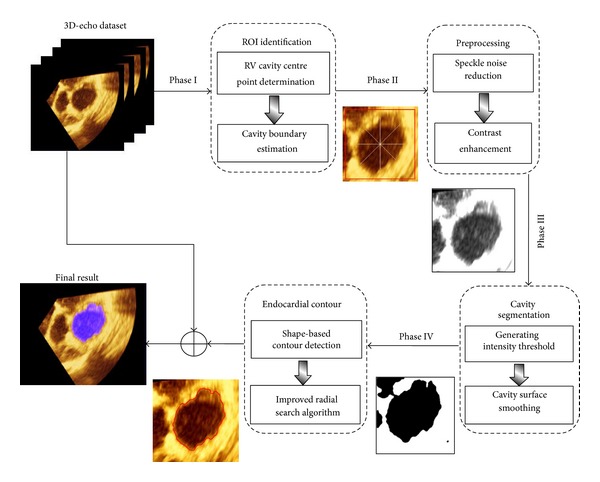
The schematic framework of the proposed method for RV endocardial contour extraction.

In this study, the main aim is to propose an accurate and robust method of the RV endocardial contour extraction over complete cardiac cycle. The greatest challenges in determining the cardiac structure of RV echocardiographic images are the thin walls, heavy trabeculation, and no simple geometric shape that approximates to the RV. Toward this aim, four processing phases are performed and each phase comprised two main stages.  Figure 2 demonstrates the overall framework of semiautomatic method for RV endocardial contour extraction.

### 3.1. Region of Interest Identification

In medical imaging, this stage is used to reduce the effort required by identifying the region of interest ROI successfully which currently is the RV cavity. The ROI identification can minimize the time needed to analyze data from image content by extracting the object in demand from an undesirable background. The complex anatomy of the RV structure causes some problems, especially when the cavity area includes the papillary muscles (i.e., these muscles are attached to the tricuspid valve and are responsible for valve movement) and the trabecular tissue. Therefore, to overcome these anatomical difficulties through ROI identification process, a new algorithm was proposed using two procedures of RV cavity centre point (RVCCP) determination and cavity boundary estimation.

#### 3.1.1. RVCCP Determination

**Figure 3:**
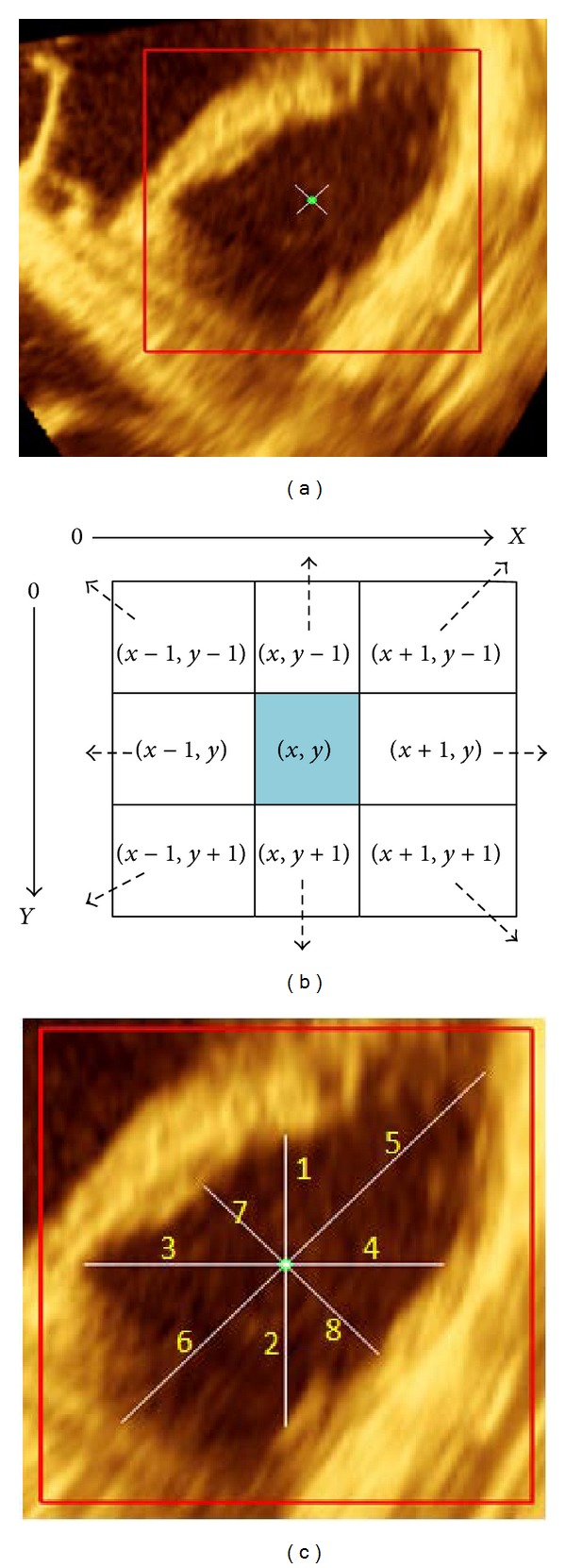
RV end-diastolic frame with initial RVCCP and initial ROI (a), RVCCP with 8-neighbours (b), and geometrical eight-distances of RV cavity diameters (c).

Due to the asymmetric shape of the RV, no standard guidelines can be followed to determine the cavity centre point over a complete cardiac cycle. Traditionally, reliable RVCCP estimation is done by experts such as cardiologists or cardiac technicians. An initial centre point *C*_*i*_ is manually marked by the expert at the end-diastolic (ED). In the context of our method, *C*_*i*_ is considered as a reference point and its (*x*, *y*) coordinates are defined in two scalars *X*_*c*_ and *Y*_*c*_. An initial ROI_*i*_ is defined around the RV cavity based on the reference centre point *C*_*i*_. This ROI_*i*_ is a square with an offset of 150-pixels from the origin. Specifically, this is defined as (*X*_*c*_ − 150, *Y*_*c*_ − 150) and (*X*_*c*_ + 150, *Y*_*c*_ + 150), as shown in Figure 3(a). Each pixel intensity *I* (*x*, *y*) within the initial ROI is saved in a new 2D matrix, and *x*, *y* are the spatial coordinates of the pixels in the new image. Then, the reference center point *C*_*i*_ and ROI_*i*_ will be subjected for next procedure of cavity boundary estimation. In addition, to determine the next RVCCP for the remaining frames, the mean value of *x*, *y* coordinates will be considered as a reference centre point for the extracted current ROI.

#### 3.1.2. Cavity Boundary Estimation

To successfully identify the remaining ROI_*s*_, an automated technique is proposed for cavity boundary estimation. It is based on the RVCCP and geometrical distance calculation (GDC) algorithm. Several steps are required and explained as follows.

*Step* *1*. Consider the current centre point $\overset{´}{C}(x,y)$ as a reference* RVCCP.*

*Step* *2*. Compute theaverage value of pixels intensities within the initial ROI_*i*_. This will be considered as the local-threshold value *L*_*T*_. 

*Step* *3*. Let $\overset{´}{p}$ be the pixel intensity value and start a loop of pixel-by-pixel comparison from the centre point $\overset{´}{C}\;(x,y)$ in eight-directions, as seen in Figure 3(b) with *L*_*T*_ value until $\overset{´}{p}$ > *L*_*T*_. 

*Step* *4*. Then, eight-points were detected and estimated to the RV inner surface boundary which will be saved as scalar ${\underline{B}}_{k}$.

*Step* *5*. Identify the geometrical distances of the RV cavity diameters from vertical (V), horizontal (H), and two-diagonal orientations (*G*_1_ and *G*_2_), respectively. All distances are computed as total number of pixels in each direction from the centre point to the estimated border point ${\underline{B}}_{k}$, and the values are saved as scalar *D*_*k*_ where *k* = 1,…, 8, as illustrated in Figure 3(c). 

*Step* *6*. Determine the maximum length of these eight geometrical distances as a constant value: *m* = {max⁡[*D*_*k*_, *D*_*k*+1_,…, *D*_8_]}. Then, *m* value will be used for ROI identification in next step. 

*Step* *7*. The new ROI is identified as a square in size from the corner at (*X*_*c*_ − *m* − 50, *Y*_*c*_ − *m* − 50) to the corner at (*X*_*c*_ + *m* + 50, *Y*_*c*_ + *m* + 50), which represents the closest area around the current RV cavity. Thus, all pixel intensities within this ROI are saved in a new image $\dot{\overset{´}{S}}$ for next processing.

**Figure 4:**
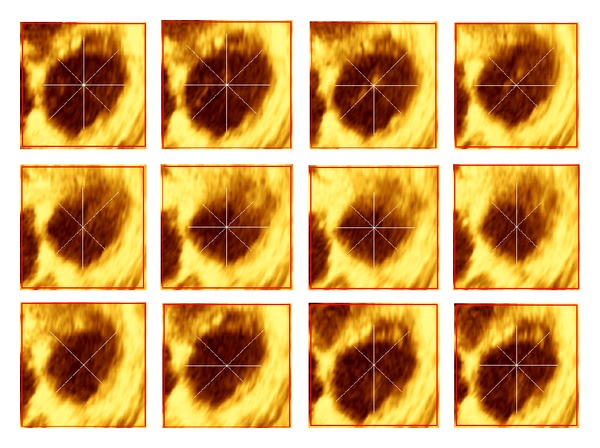
Automatic ROI identification of 1st slice through complete cardiac cycle.

As mentioned earlier, the complex anatomy of the RV cavity structure causes two main problems, the papillary muscles and the trabecular tissue. Despite these cavity anatomical complexities, the ROI of RV cavity over complete cardiac cycle has been determined accurately and precisely as presented in Figure 4.

### 3.2. Pre-Processing

A preprocessing phase is one of the fundamental issues in the field of medical image analysis. It is very important to obtain accurate observation for feature extraction, recognition, and quantitative measurements. Therefore, the principle objective of preprocessing is to increasing the consistency and reliability for clinical measurements. For this purpose, two processing tasks were implemented on gray-scale of image $\dot{\overset{´}{S}}$ for speckle noise reduction and contrast enhancement.

#### 3.2.1. Speckle Noise Reduction

In this work, Gaussian filter has been used as a smoothing operator and performs a weighted average of surrounding pixels based on the Gaussian distribution. The Gaussian operator generates a matrix of matrix of values *G*(*x*, *y*) that are applied togroups of pixels in the image. These matrix values can be defined by the following 2D Gaussian equation:

(1) $$\begin{array}{c} G\left(x,y\right)=\frac{1}{2\pi \sigma^{2}}\exp\left\{-\frac{x^{2}+y^{2}}{2\sigma^{2}}\right\}, \end{array}$$

where Sigma *σ* is the standard deviation of the distribution and defines the amount of blurring and also the degree of smoothing. The filtered image could be even smoother when high sigma *σ* values are used, but the difficulty is that it requires significantly more calculations per pixel.

#### 3.2.2. Contrast Enhancement

**Figure 5:**
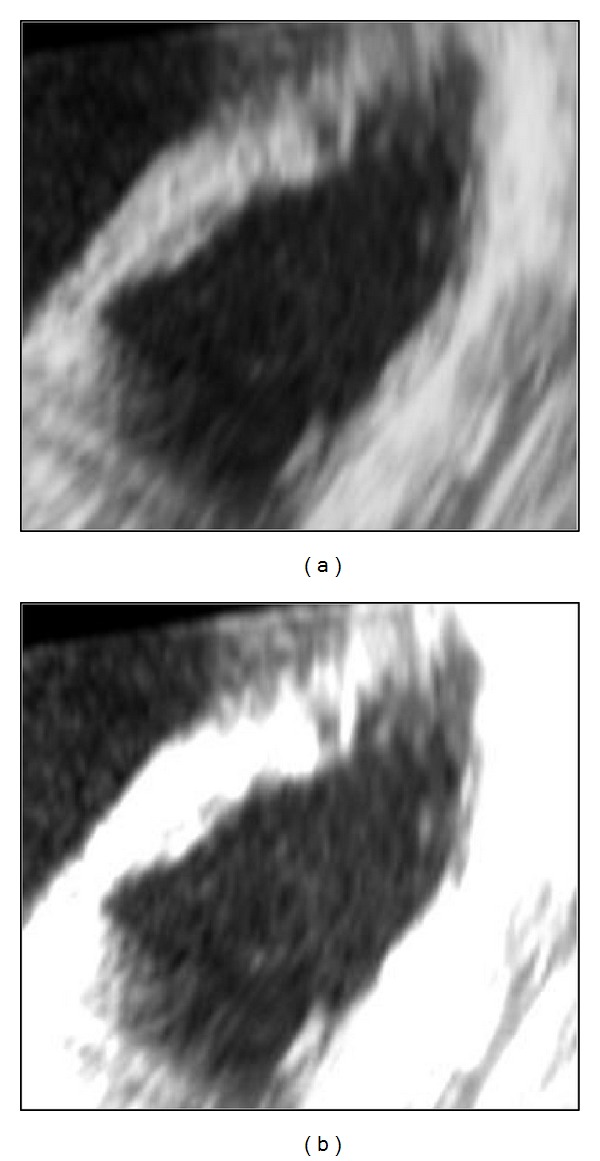
Smoothed image by Gaussian filter (a) and contrast enhanced image by improved method (b).

The main aim of contrast enhancement in echocardiography images is to improve the quality and appearance of an image for visual interpretation and gain a better understanding of the imagery. There are a variety of image enhancement methods based on pixel and local image enhancement operations. The percentage linear stretch contrast is one method of point operations and is used for highlighting the regions of interest. In this study, this method was improved by generating a contrast factor automatically based on local variation of pixels intensities according to the mean and standard deviation values. Hence, the contrast factor is capable of controlling the contrast adjustment percentage of image intensity values. More specifically, it is used to highlight the myocardial wall tissue and darken the RV cavity region by manipulating the image brightness. Practically, all pixel intensity values in an input image are modified into new values in an output image independently using

(2) $$\begin{array}{c} h\left(i,j\right)=\text{INT}\left\{\frac{255}{\left(\mathrm{Max}-\mathrm{Min}\right)}*\left[G\left(i,j\right)-\mathrm{Min}\right]\right\}, \end{array}$$

where *G*(*i*, *j*) are the grey level intensitiesinthe smoothed image and *h*(*i*, *j*) is the output result of the new pixel intensity that is normalized in the range (0–255). Two values, Min and Max, refer to the minimum and maximum specified as the new values in the output range using the following equations:

(3a) $$\begin{array}{c} \mathrm{Min}=\mu -\beta *\partial , \end{array}$$

(3b) $$\begin{array}{c} \mathrm{Max}=\mu +\beta *\partial , \end{array}$$

where *μ* and ∂ are the mean value and the standard deviation of all pixel intensity values. The factor *β* is the contrast ratio percentage, which equals 1.5 in our experiments to control the percentage of contrast improvement. Figures 5(a) and 5(b) depict an example of experimental results for smoothed image and the contrast enhanced image, respectively.

### 3.3. Cavity Segmentation

In recent years, image segmentation task has presented an important and challenging issue for most researchers in the field of diagnostics concerning heart abnormalities. In echocardiography, the main objective in most methods is to develop the segmentation process by distinguishing the image into distinct regions—blood pool (i.e., ventricular cavity) and myocardial wall. One of the most complex anatomical problems that can be observed in the RV cavity region is the heavily trabeculated wall which makes automatic segmentation of RV cavity a difficult task. Therefore, this Phase III includes two processing stages: generating intensity threshold and cavity surface smoothing.

#### 3.3.1. Generating Intensity Threshold

One of the most common segmentation methods is thresholding, which used to separate the image with bimodal histogram into a foreground object region from the background. The basic key here is a threshold value which is generated for once in all frames that belongs to individual 3D dataset video. We use the $\dot{\overset{´}{S}}$ matrix of the extracted ROI gradients to generate threshold value *τ* which is equal to the mean value of pixel intensities as follows:

(4) $$\begin{array}{c} \tau_{\text{threshold}}=\frac{\sum_{i=1}^{N}\sum_{j=1}^{N}\dot{\overset{´}{S}}\left(i,j\right)}{N^{2}}. \end{array}$$

Hence, a comparison operation was done automatically to each pixel's intensity value with threshold value *τ*. The resultant binary image *S*(*i*, *j*) was segmented into two regions, the white pixels with value 1 s or other convenient intensity level (i.e., 255) that correspond to the myocardial wall tissue, whereas the black pixels with value 0s correspond to the RV cavity.

#### 3.3.2. Cavity Surface Smoothing

This stage is very important for two reasons: firstly, to fill gaps between muscle tissues that may appear within the cavity area, secondly, to improve the RV cavity shape by smoothing the inner surface border that is endocardium wall. Therefore, two morphological operators “erosion” and “dilation” were applied on the resultant binary image with structuring elements of size 3 × 3 pixels. In more detail, the erosion of *A* by the structuring element *B* is defined by *A*Θ*B*, and the dilation of *A* by the structuring element *B* is denoted by *A* ⊕ *B*. Hence, the morphological opening process is denoted by *AоB*, which is obtained from the erosion followed by dilation of the resulting image by B, as identified as follows:

(5) $$\begin{array}{c} AоB=\left(A\Theta B\right)⊕B. \end{array}$$

Accordingly, in each erosion step of *A*, if any neighbour pixel is white then the current pixel is set to white. Likewise, in each dilation step of *B*, if any neighbour pixel is black then the current pixel is set to black [33]. Note that in our experiments, three iterations of erosion followed by two iterations of dilation were found to be effective in obtaining good and accurate segmentation results for RV cavity.

### 3.4. Endocardial Contour Extraction

In echocardiography imaging, automatic extraction of the RV endocardial contour would improve the reliability of the quantitative analysis by eliminating manual tracing. The main goal is to automatically detect and extract the RV endocardial contour over a complete cardiac cycle through 4-slices of 3D echocardiography. Towards this goal, a robust hybrid method is proposed by combining a shape-based contour detection and an improved radial-search algorithm. This is an important contribution to this work, especially when RV endocardial contour is extracted accurately to be a useful diagnostic tool for assessing the RV systolic function qualitatively and quantitatively. The resultant binary-segmented image of RV cavity from the previous Phase III will be used to be the input image for this Phase IV.

#### 3.4.1. Shape-Based Contour Detection

**Figure 6:**
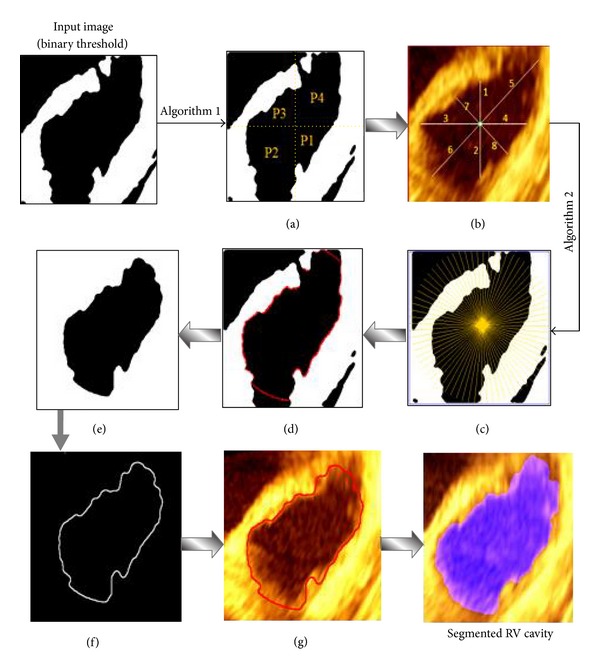
The schematic framework of implementation steps for the proposed hybrid method for RV endocardial contour extraction.

A shape-based contour detection algorithm is proposed to determine the maximum distance of the RV cavity diameters for each of the four parts individually. Therefore, the proposed algorithm (Algorithm 1) comprises two processing steps. The first step focuses on partitioning the RV cavity area into four parts: P1, P2, P3, and P4 according to the asymmetric shape as seen in Figure 6(a). The second step was applied to compute the maximum distance of the RV cavity diameters in each part based on geometrical distances calculations from current RV cavity centre point to the inner surface boundary points as shown in Figure 6(b). Then, these four maximum distances will be used as input parameters for the next task of RV endocardial contour extraction. The pseudocode of shape-based contour detection is presented in Algorithm 1.

#### 3.4.2. Improved Radial Search Algorithm

The main objective of this stage is to accurately extract the RV cavity from the background by estimating the actual boundary of the endocardial wall. The radial search algorithm was improved based on the maximum distance value of each part in the RV cavity. It works through circularly scanning the ROI in clockwise fashion to detect the RV cavity boundary. Accordingly, the RV cavity is segmented from the background according to the detected contour points. In order to compute the new RVCCP, the mean value of the *x*-*y* coordinates for segmented RV cavity area is calculated and considered as the new cavity centre point. Thus, this new RVCCP will be used as the reference centre point for the RV cavity in the next frame. The improved radial research algorithm largely depends on the precise results of edge detection for RV endocardial. Finally, the extracted endocardial contour is superimposed on the original 3D echocardiography image. The implementation steps of the improved radial search algorithm (Algorithm 2) based on maximum distance for each part of RV cavity which is obtained from Algorithm 1 are illustrated in Algorithm 2.

The proposed hybrid method implementation will be repeated for all frames through the 4-slices. The resultant images for each slice are stored in specific folders individually. Figure 6 demonstrates the schematic framework for the overall implementation steps of proposed hybrid method.

## 4. Experimental Results 

**Figure 7:**
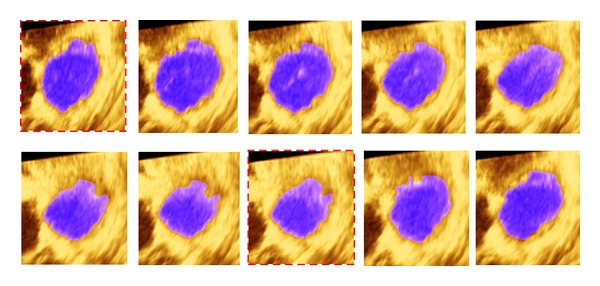
The final results of segmented RV cavities based on endocardial contour extraction over complete cardiac cycle with red border labelled at ED and ES, respectively.

In this study, all datasets were used from a Malaysian hospital, IJN, using the “Philips” medical system with a matrix array X2-7t transducer (TEE). Sixteen datasets pertaining to four patients were acquired from 3D echocardiography full volume encompassing the right ventricle in 4-slices of long-axis view. Two datasets have a clear RV cavity unlike the other two, which have a complex anatomy of the RV cavity including papillary muscle and trabecular tissue. Accordingly, the proposed approach was implemented using echocardiography videos (i.e., four slices in long-axis view per patient with total frames of 216). The spatial dimensions for each image are 1024 × 760 pixels in width and height, respectively. The accuracy of the experimental results obtained by our semiautomatic method for RV cavity segmentation depends on whether the true endocardial contour was extracted successfully and effectively over a cardiac cycle. Moreover, the RV cavity area was considered as the total number of pixels within the cavity region based on the extracted RV endocardial contour. Then, the maximum and minimum areas were determined automatically and, respectively, considered as end-diastolic area (EDA) and end-systolic area (ESA). In Figure 7, the experimental results by the proposed method for the segmented RV cavities are presented using 1st slice (patient number 1) from 3D dataset and labelled with dashed red border at EDA and ESA, respectively.

As mentioned in Section 1, manual tracing for RV endocardial contour through a complete cardiac cycle is a tedious and time consuming task which often requires several minutes to perform for each frame. In many cases, time is a crucial factor when assessing the ventricular function abnormality based on specialist decisions. Two examples of clear and complex RV anatomy from 3D dataset in 4-slices with a total of 80-frames are being traced.  Table 1 illustrates the time taken for manual tracing the RV endocardial contour by the specialist without adjustments racing for each dataset. It can be noted from Table 1 that the time required varies according to the anatomical complexity of the dataset as well as the total number of frames in each 3D dataset. On the other hand, the processing time for automatic-tracing using the proposed method was approximately 12 seconds per frame, thereby saving the processed image for each stage, and could be less when the RV cavity area has a greater clarity in the tissue structure. Therefore, the automatic-tracing of RV endocardial contour by the proposed method is faster than manual-tracing method and highly desirable to support the assessment processes of RV function abnormality, in particular with accurate and reliable results.

### 4.1. Quantitative Evaluation Measures

The performance of the proposed semiautomatic method depends on the successful extraction results for the RV endocardial contour over a complete cardiac cycle. In our work, the experimental results of RV endocardial contour extraction were evaluated quantitatively by comparing the automated results through all frame sequences versus the ground truth results which were manually marked/annotated by the specialist (Consultant Cardiac Anesthesiologist). Therefore, four quantitative metrics were used and explained in details as follows.

**Figure 8:**
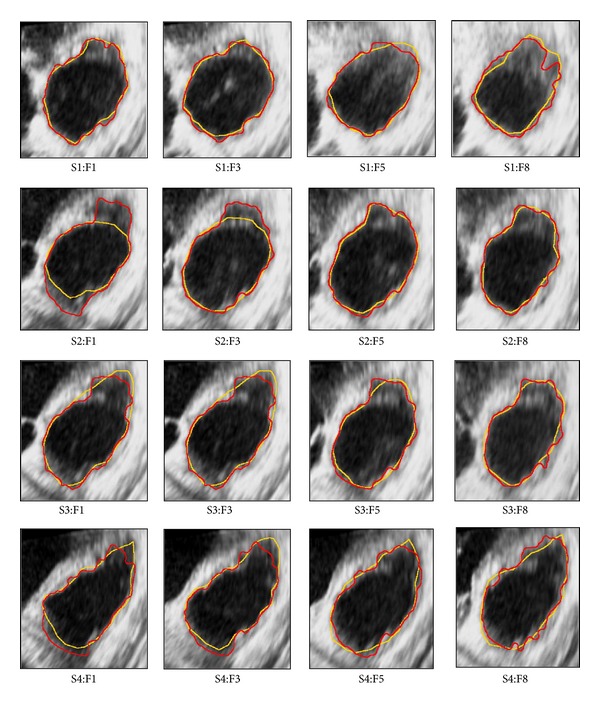
The results of paired-contours of RV endocardial in 4-slices at different frames over complete cardiac cycle by manual-traced (yellow) and automatic-extracted (red).

(1) Root mean square distance (RMSD) measures the root mean square distances between paired contour points [34]. Here, it is used to compare the experimental results of the RV endocardial contour that is obtained by the proposed automatic extraction method with those obtained from the manually traced contours (ground truth) by the specialist.  Figure 8 demonstrates several examples of paired manual-to-automatic results through 4-slices at different frames over a complete cardiac cycle.

For both methods, the RV contour points are saved in two scalars *C* and $\overset{´}{Ç}$, respectively. In this context, the RMSD was measured based on the distance calculation as in the following formula:

(6) $$\begin{array}{c} \text{RMSD}=\sqrt{{\frac{1}{N}}^{\;}\overset{N}{\underset{i=1}{\sum }}\text{distance}\left(C_{i},{\overset{´}{Ç}}_{i}\right)}, \end{array}$$

where *N* is the length in pixels of each contour which is equal to 360 points in our implementation. Each contour point has *x*, *y* coordinates as {(*x*_*j*,1_, *y*_*j*,1_), (*x*_*j*,2_, *y*_*j*,2_), …, (*x*_*j*,*n*_, *y*_*j*,*n*_)}, where the distance has been calculated by Euclidian distance using the following formula:

(7) $$\begin{array}{c} \text{distance}\left({C_{i}}_{},{\overset{´}{Ç}}_{i}\right)=\left({xC}_{i}-x{\overset{´}{Ç}}_{i}\right)²+\left({yC}_{i}-y{\overset{´}{Ç}}_{i}\right)². \end{array}$$

**Figure 9:**
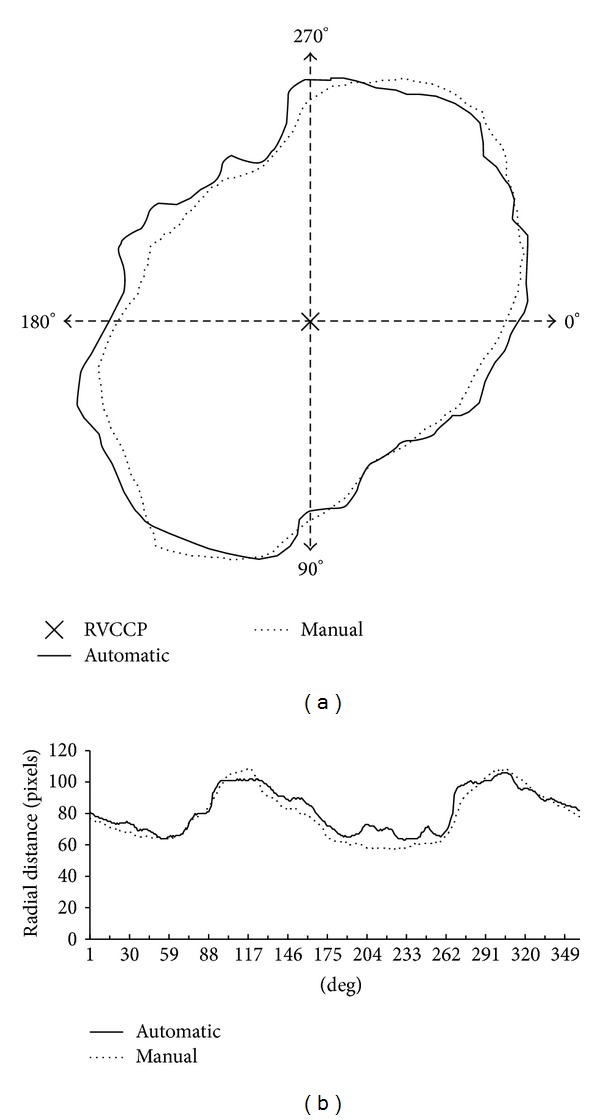
An example of manual-to-automatic contour extraction (a) and the comparative analysis based on the radial distances from RVCCP (b).

Figure 9(a) depicts an example of the manual-to-automatic contours with identified RVCCP. The comparative analysis of the paired contours are done using the polar format based on the radial distances started from 0° to 360° for each contour as shown in Figure 9(b).

(2) Dice similarity coefficient (DSC) is one of the common metrics used for evaluating the segmentation results by comparing the area of the segmented object against the reference. In particular, it measures the overlap between two segmented areas of the RV cavity. Consider that *R*_*M*_ is the ground truth area that is manually drawn by the specialist and *R*_*A*_ is the area obtained automatically by our proposed method; then the measure is given as follows:

(8) $$\begin{array}{c} \text{DSC}\left(R_{M},R_{A}\right)=\frac{2\left(R_{M}\cap R_{A}\right)}{R_{M}+R_{A}}, \end{array}$$

where ∩ represents the intersection between the two overlapping areas of RV cavity regions *R*_*M*_ and *R*_*A*_. The Dice coefficient gives a measure value between 0 (no overlap “total mismatch”) and 1 (full overlap “perfect match”) [35]. Based on the DSC value, the similarity between the *R*_*M*_ and *R*_*A*_ can be defined as a percentage.

(3) Area error metrics are used for measuring the area error of the segmented RV cavities by comparing the results of our proposed automated method *Ω*_*A*_ versus the ground truth method *Ω*_*B*_. The RV cavity area *Ω*_*B*_ for each frame in a complete cardiac cycle has been measured by “area plug-in” function from QLAB software using manual tracing of the endocardial contour by the specialist. This process has been repeated for the entire 3D echocardiography datasets used for comparative analysis. Hence, two measures were used, namely, percentage of true-positive area (PTP) and percentage of false-positive area (PFP), which can be defined as follows [36]:

(9) PTP=Area(ΩA∩ΩB)Area(ΩA)%,PFP=Area(ΩA)−Area(ΩA∩ΩB)Area(ΩB)%.

**Figure 10:**
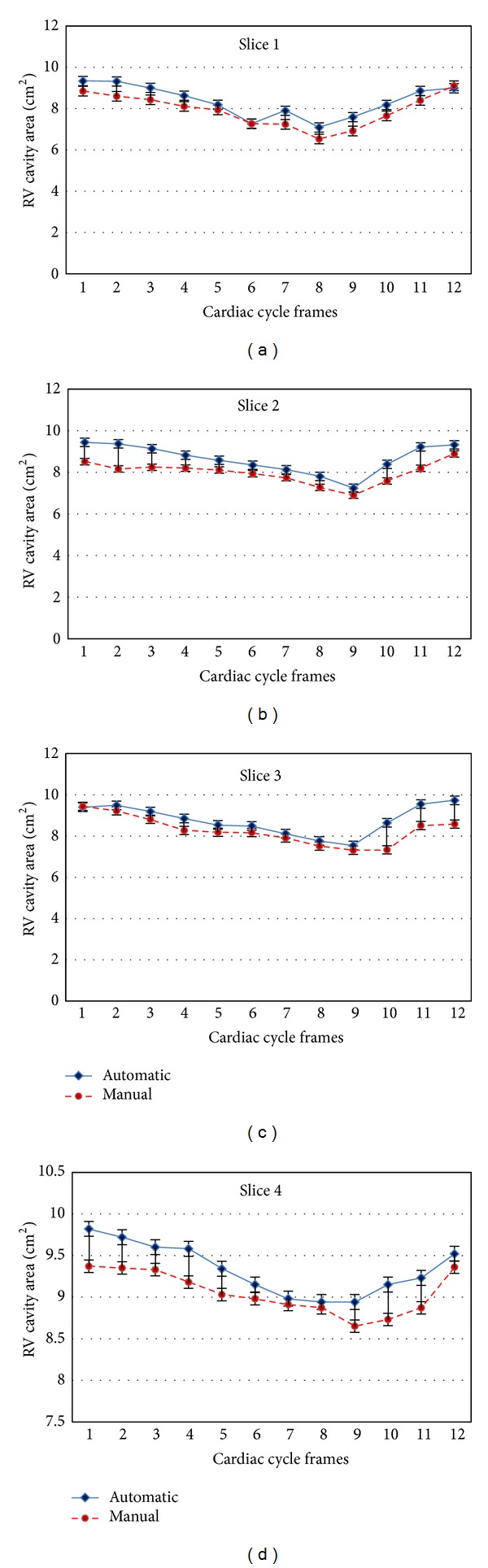
The comparative analysis of RV cavity area measurements in 4-slices through complete cardiac cycle using automatic and manual methods.

**Figure 11:**
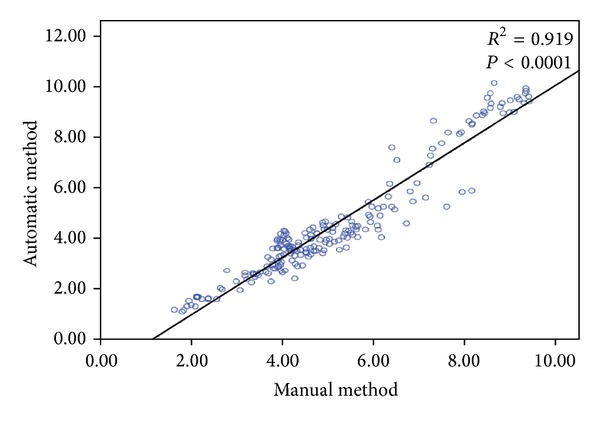
Linear regression plot of comparative analysis for RV cavity area measured in cm^2^ by manual and automatic methods.

Table 2 summarizes the comparison analysis for the proposed method performance using these four quantitative measures (RMSD, DSC, PTP, and PFP), which are presented in terms of Mean ± SD. It can be noted the set number 1 for all slices have the higher values of PFP among other dataset. In particular, slice 2 from this dataset has the maximum value 11.37 ± 6.30 of PFP. This result has been obtained due to inaccurate manual tracing for RV endocardial contour of some frames. For the overall dataset, the distance error of the RV endocardial contour in the paired manual-to-automatic by RMSD measure was 2.21 ± 0.35 (mm) and the RV cavity area has 87% similarity with DSC. The PTP results show that the proposed approach performs efficiently in all dataset with overall performance of 95%. The statistical comparative analysis with standard error bars for the RV cavity area measurements (in cm^2^) through 4-slices between the proposed method *R*_*A*_ and the manual method *R*_*M*_ is presented in Figure 10. Furthermore, the linear regression analysis between the two methods—*manual *and* automatic *is demonstrated in Figure 11. The total 3D echocardiography images used are 216 for RV area measurements in both method that indicated by blue circles. It can be noted for both methods, the spread of the values is relatively low, demonstrating that inaccurate segmentation results does not have much influence in the RV area measurements. It can also be seen from figure, the regression coefficient is good and the comparative analysis showed a close relationship (*r* = 0.92) between manual and automatic methods.

## 5. Discussion and Conclusion

In this paper, a robust method of RV endocardial contour extraction was presented through four major processing phases on 3D echocardiography dataset. The overall framework presents a semiautomatic method for RV endocardial contour extraction with very minmal user interaction required, except for selecting the initial RV cavity centre point at first frame, that is, end-diastolic. The RV endocardial contour in all frames was extracted automatically using a robust combination of shape-based border detection and improved radial search algorithm. It should also be noted that this hybrid method is independent for extracting the RV endocardial contour without prior shape knowledge over a complete cardiac cycle. One limitation is that it did not show optimum results for RV cavity segmentation in special cases with poor image quality and heavy trabeculation of the RV wall tissue. The proposed method has been implemented efficiency for 3D echocardiography dataset using a computer, Microsoft Windows 7 with 4-GHz CPU speed. Thus, as mentioned earlier, the computation time is approximately 12 s for each frame, thereby saving the processed image for each stage. On the other hand, further quality improvements of acquired images might yield more accurate and reliable segmentation results for RV cavities, especially in the comparative analysis over a complete cardiac cycle. Although our proposed method was applied on 4-slices per patient, which were cropped automatically using the “2 × 2 iSlices” plug-in by QLAB software, it is also possible to obtain 3 × 3 or 4 × 4 slices; this is planned for future work.

The proposed method comprises four major phases of image processing and analysis. The combination of these steps is the main contribution of the proposed work. The ROI, that is, the closest region of the RV cavity area, was determined precisely through an automatic process based on the cavity centre point identification and geometrical distance calculation. The proposed method works well despite the anatomical complexity for RV cavity with papillary muscles. Hence, the ROI of RV cavity has been determined accurately and precisely through complete cardiac cycle. The contrast enhancement is important step in our contribution because it is done automatically once to all dataset based on the percentage linear stretch method. This method was improved to control the image brightness by generating a contrast factor. In addition, the RV cavity area was segmented using the unique threshold value, which was generated automatically in the same way for all datasets, unlike most segmentation methods that need to use iterative processes for generating the desired threshold values. The overall results of the comparative analysis showed how important it is to extract the RV endocardial contour accurately to obtain more precise results, which might be a useful diagnostic tool for assessing the RV systolic function qualitatively and quantitatively. The experimental results accuracy of proposed method has 95% of PTP and has 87% similarity to the ground truth results. The distance error measure of RMSD for RV endocardial contour in terms of mean ± SD was found to be 2.21 ± 0.35 mm. Therefore, our proposed method can be considered as a robust method for RV endocardial contour extraction, and, to our knowledge, in recent research it is the first work to extract the RV endocardial contour and to automate the use of 3D echocardiography full volume dataset in the field of CAD. Therefore, automated contour extraction would improve the reliability of the quantitative analysis by eliminating the subjectivity of manual tracing. In particular, it could also be a significant prognostic factor in regional ventricle abnormality, congenital heart disease, and cardiac surgery.

## Figures and Tables

**Algorithm 1:**
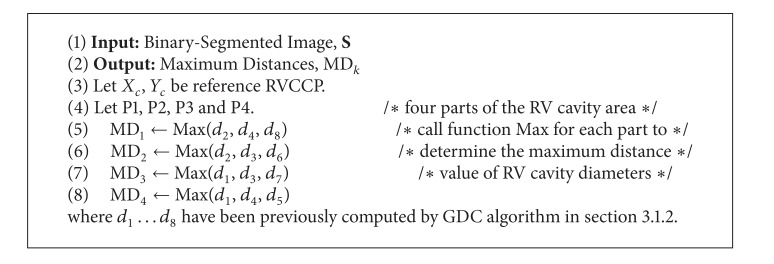
Shape-based contour detection.

**Algorithm 2:**
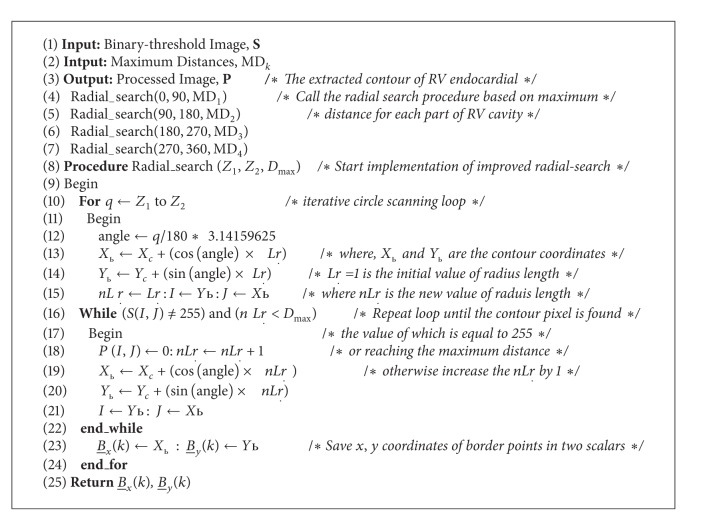
Improved radial search method.

**Table 1:** Total time taken in minutes:seconds to manually trace the RV endocardial contours.

Dataset of 3D echo images *N* = 80	Slice 1	Slice 2	Slice 3	Slice 4	Total time
Set number 1 Clear RV cavity	5:28 (*n* = 12)	4:35 (*n* = 12)	3:42 (*n* = 12)	3:00 (*n* = 12)	16:05 (*n* = 48)
Set number 2 Complex RV cavity	6:02 (*n* = 8)	5:30 (*n* = 8)	5:00 (*n* = 8)	3:55 (*n* = 8)	20:27 (*n* = 32)

**Table 2:** Quantitative evaluations for experimental results of RV endocardial contour extraction using manual-to-automatic comparison.

3D-echo data Total *n* = 216	4-slices	RMSD	DC	PTP (%)	PFP (%)
Set number 1 (*n* = 48)	S1	1.62 ± 0.24	0.94 ± 0.02	92.20 ± 3.17	8.40 ± 3.90
	S2	1.81 ± 0.30	0.94 ± 0.03	89.90 ± 6.30	11.37 ± 6.30
	S3	2.02 ± 0.41	0.92 ± 0.02	90.32 ± 3.55	10.45 ± 4.46
	S4	2.29 ± 0.42	0.92 ± 0.01	90.70 ± 3.10	9.83 ± 3.82

Set number 2 (*n* = 68)	S1	2.54 ± 0.38	0.88 ± 0.03	97.85 ± 0.54	1.80 ± 0.56
	S2	3.16 ± 0.29	0.83 ± 0.02	98.19 ± 0.31	1.35 ± 0.26
	S3	2.36 ± 0.32	0.86 ± 0.02	97.92 ± 1.11	1.71 ± 1.14
	S4	2.34 ± 0.38	0.88 ± 0.03	97.80 ± 0.58	1.82 ± 0.60

Set number 3 (*n* = 56)	S1	2.25 ± 0.39	0.80 ± 0.04	97.40 ± 1.60	2.04 ± 1.49
	S2	2.43 ± 0.30	0.83 ± 0.02	98.30 ± 0.29	1.27 ± 0.26
	S3	2.74 ± 0.28	0.80 ± 0.05	98.35 ± 0.26	1.20 ± 0.31
	S4	2.82 ± 0.43	0.82 ± 0.03	98.21 ± 0.27	1.30 ± 0.28

Set number 4 (*n* = 44)	S1	1.30 ± 0.21	0.92 ± 0.01	96.87 ± 1.01	2.86 ± 0.99
	S2	1.78 ± 0.20	0.89 ± 0.02	94.70 ± 3.34	5.51 ± 3.51
	S3	2.07 ± 0.24	0.87 ± 0.02	92.75 ± 3.73	6.78 ± 3.79
	S4	1.93 ± 0.28	0.88 ± 0.03	91.45 ± 4.05	8.45 ± 4.49

Overall		2.21 ± 0.35	0.87 ± 0.026	95.59 ± 1. 75	4.37 ± 1.94

## References

[1] Stefanadis CI (2010). Imaging of the neglected cardiac chamber: the right ventricle. *Hellenic Journal of Cardiology*.

[2] Selton-Suty C, Juillière Y (2009). Non-invasive investigations of the right heart: how and why?. *Archives of Cardiovascular Diseases*.

[3] Helbing WA (2004). Right ventricular function: the comeback of echocardiography?. *European Journal of Echocardiography*.

[4] Triantafyllou K, Kranidis A, Karabinos E, Grassos H, Babalis D (2010). Clinical implications of the echocardiographic evaluation of right ventricular function on the long axis using newer techniques. *Hellenic Journal of Cardiology*.

[5] Blyth KG, Peacock AJ (2009). Imaging the right ventricle in pulmonary hypertension. *PVRI Review*.

[6] Hesse B, Asher CR (2005). Time to move to the right: the study of right ventricular systolic performance: too long neglected. *Clinical Cardiology*.

[7] Noble JA, Boukerroui D (2006). Ultrasound image segmentation: a survey. *IEEE Transactions on Medical Imaging*.

[8] Rogowska J, Bankman IN (2008). Overview and fundamentals of medical image segmentation. *Handbook of Medical Image Processing and Analysis*.

[9] Mitchell SC, Lelieveldt BPF, van der Geest RJ, Bosch HG, Reiber JHC, Sonka M (2001). Multistage hybrid active appearance model matching: segmentation of left and right ventricles in cardiac MR images. *IEEE Transactions on Medical Imaging*.

[10] Liu N, Crozier S, Wilson S Right ventricle extraction by low level and model-based algorithm.

[11] Katouzian A, Prakash A, Konofagou E A new automated technique for left- and right-ventricular segmentation in magnetic resonance imaging.

[12] Carminati MC, Gripari P, Maffessanti F Semi-automated border detection for right ventricular volume estimation from MR images.

[13] Souto M, Masip LR, Couto M (2013). Quantification of right and left ventricular function in cardiac MR imaging: comparison of semiautomatic and manual segmentation algorithms. *Diagnostics*.

[14] Dawood FAA, Rahmat RW, Dimon MZ, Nurliyana L, Kadiman SB (2011). Automatic boundary detection of wall motion in two-dimensional echocardiography images. *Journal of Computer Science*.

[15] Rahmat RW, Dawood FA, Kadiman SB, Abdullah LN, Zamrin MD Border detection of ventricle wall motion in echocardiographic images: a survey.

[16] Angelini ED, Homma S, Pearson G, Holmes JW, Laine AF (2005). Segmentation of real-time three-dimensional ultrasound for quantification of ventricular function: a clinical study on right and left ventricles. *Ultrasound in Medicine and Biology*.

[17] Ostenfeld E, Carlsson M, Shahgaldi K, Roijer A, Holm J (2012). Manual correction of semi-automatic three-dimensional echocardiography is needed for right ventricular assessment in adults; Validation with cardiac magnetic resonance. *Cardiovascular Ultrasound*.

[18] Salvador A, Maingourd Y, Fu S, Lerallut JF Optimization of an edge detection algorithm for echocardiographic images.

[19] Zagrodsky V, Walimbe V, Castro-Pareja CR, Qin JX, Song J-M, Shekhar R (2005). Registration-assisted segmentation of real-time 3-D echocardiographic data using deformable models. *IEEE Transactions on Medical Imaging*.

[20] Pickard JE, Hossack JA, Acton ST Shape model segmentation of long-axis contrast enhanced echocardiography.

[21] Orderud F, Rabben SI Real-time 3D segmentation of the left ventricle using deformable subdivision surfaces.

[22] Nandagopalan S, Adiga BS, Dhanalakshmi C, Deepak N Automatic segmentation and ventricular border detection of 2D echocardiographic images combining K-means clustering and active contour model.

[23] Haddad F, Couture P, Tousignant C, Denault AY (2009). The right ventricle in cardiac surgery, a perioperative perspective: I. Anatomy, physiology, and assessment. *Anesthesia and Analgesia*.

[24] Wahl A, Praz F, Schwerzmann M (2011). Assessment of right ventricular systolic function: comparison between cardiac magnetic resonance derived ejection fraction and pulsed-wave tissue Doppler imaging of the tricuspid annulus. *International Journal of Cardiology*.

[25] Jurcut R, Giusca S, la Gerche A, Vasile S, Ginghina C, Voigt J-U (2010). The echocardiographic assessment of the right ventricle: what to do in 2010?. *European Journal of Echocardiography*.

[26] Lacerda SG, da Rocha AF, Vasconcelos DF, de Carvalho JLA, Sene IG, Camapum JF Left ventricle segmentation in echocardiography using a radial-search-based image processing algorithm.

[27] McCulloch ML, Little SH (2009). Imaging methodology and protocols for three-dimensional echocardiography. *Current Opinion in Cardiology*.

[28] Fischer GW, Salgo IS, Adams DH (2008). Real-time three-dimensional transesophageal echocardiography: the matrix revolution. *Journal of Cardiothoracic and Vascular Anesthesia*.

[29] Shiota T (2009). 3D echocardiography: evaluation of the right ventricle. *Current Opinion in Cardiology*.

[30] Chua S, Levine RA, Yosefy C (2009). Assessment of right ventricular function by real-time three-dimensional echocardiography improves accuracy and decreases interobserver variability compared with conventional two-dimensional views. *European Journal of Echocardiography*.

[31] Lang RM, Badano LP, Tsang W (2012). EAE/ASE recommendations for image acquisition and display using three-dimensional echocardiography. *Journal of the American Society of Echocardiography*.

[32] Leung KYE, Bosch JG (2010). Automated border detection in three-dimensional echocardiography: principles and promises. *European Journal of Echocardiography*.

[33] Sonka M, Hlavac V, Boyle R (2007). *Image Processing, Analysis, and Machine Vision*.

[34] MeloJúnior SA, Macchiavello B, Andrade MM (2010). Semi-automatic algorithm for construction of the left ventricular area variation curve over a complete cardiac cycle. *BioMedical Engineering Online*.

[35] Grosgeorge D, Petitjean C, Caudron J, Fares J, Dacher J-N (2011). Automatic cardiac ventricle segmentation in MR images: a validation study. *International Journal of Computer Assisted Radiology and Surgery*.

[36] Zhu Y, Papademetris X, Sinusas A, Duncan J (2012). Automated segmentation of real-time 3D echocardiography using an incompressibility constraint. *Echocardiography—New Techniques*.

